# The prevalence and spectrum of reported incidental adrenal abnormalities in abdominal computed tomography of cancer patients: The experience of a comprehensive cancer center

**DOI:** 10.3389/fendo.2022.1023220

**Published:** 2022-11-10

**Authors:** Aiham Qdaisat, Sara Bedrose, Obadah Ezzeldin, Ahmed W. Moawad, Sai-Ching J. Yeung, Khaled M. Elsayes, Mouhammed Amir Habra

**Affiliations:** ^1^ Department of Emergency Medicine, The University of Texas MD Anderson Cancer Center, Houston, TX, United States; ^2^ Section of Endocrinology, Diabetes and Metabolism, Baylor College of Medicine, Houston, TX, United States; ^3^ Department of Diagnostic Radiology, The University of Texas Medical Branch, Galveston, TX, United States; ^4^ Department of Abdominal Imaging, The University of Texas MD Anderson Cancer Center, Houston, TX, United States; ^5^ Department of Endocrine Neoplasia and Hormonal Disorders, The University of Texas MD Anderson Cancer Center, Houston, TX, United States

**Keywords:** adrenal, incidental, cancer, abnormalities, imaging, computed tomography, abdominal, radiology report

## Abstract

**Background:**

The increasing use of computed tomography (CT) has identified many patients with incidental adrenal lesions. Further evaluation of these lesions is often dependent on the language used in the radiology report. Compared to the general population, patients with cancer have a higher risk for adrenal abnormalities, yet data on the prevalence and type of incidental adrenal lesions reported on radiologic reports in cancer patients is limited. In this study, we aimed to determine the prevalence and nature of adrenal abnormalities as an incidental finding reported on radiology reports of cancer patients evaluated for reasons other than suspected adrenal pathology.

**Methods:**

Radiology reports of patients who underwent abdominal CT within 30 days of presentation to a tertiary cancer center were reviewed and analyzed. We used natural language processing to perform a multi-class text classification of the adrenal reports. Patients who had CT for suspected adrenal mass including adrenal protocol CT were excluded. Three independent abstractors manually reviewed abnormal and questionable results, and we measured the interobserver agreement.

**Results:**

From June 1, 2006, to October 1, 2017, a total of 600,399 abdominal CT scans were performed including 66,478 scans obtained within 30 days of the patient’s first presentation. Of these, 58,512 were eligible after applying the exclusion criteria. Adrenal abnormalities were identified in 7,817 (13.4%) reports, with adrenal nodularity (3,401 [43.5%]), adenomas (1,733 [22.2%]), and metastases (1,337 [17.1%]) being the most reported categories. Only 10 cases (0.1%) were reported as primary adrenal carcinomas and 2 as pheochromocytoma. Interobserver agreement using 300 reports yielded a Fleiss kappa of 0.893, implying almost perfect agreement between the abstractors.

**Conclusions:**

Incidental adrenal abnormalities are commonly reported in abdominal CT reports of cancer patients. As the terminology used by radiologists to describe these findings greatly determine the subsequent management plans, further studies are needed to correlate some of these findings to the actual confirmed diagnosis based on hormonal, histological and follow-up data and ascertain the impact of such reported findings on patients’ outcomes.

## Introduction

In the era of advanced imaging, our ability to detect very small adrenal lesions has led to a growing number of adrenal incidentalomas, which are adrenal lesions discovered unexpectedly on an imaging test that is performed for a clinical problem unrelated to adrenal disease (1, 2). Adrenal incidentalomas are reported in 4.2%–7.3% of all abdominal computed tomography (CT) scans (3–5), which is very similar to the reported prevalence of adrenal incidentalomas on autopsy (2%-6%) (6, 7). Because studies of adrenal incidentalomas have mainly focused on the general population, the prevalence and nature of cancer patients’ adrenal incidentalomas on CT scans are not well characterized.

The workup on an adrenal lesion includes the evaluation of its hormonal function as well as its malignant potential (5, 6). Only obviously benign adrenal lesions (eg, lipid-rich adenoma, simple cyst, and myelolipoma) or obvious local malignant invasion can be definitively characterized by CT. However, CT reports can provide valuable clues to the presence of an adrenal abnormality and any malignant potential, based on its radiological appearance. Studies have shown that following the guidelines for the management of adrenal incidentaloma is influenced by the recommendations in the radiology report (8). The specific characteristics and description influence further workup and the approach to patients with adrenal incidentalomas (8).

Despite increasing data, adrenal incidentalomas still represent a public health challenge. Owing to lack of prospective studies that provide level-one, evidence-based recommendations, there is no consensus on the best management of adrenal incidentalomas. Managing these lesions in patients with cancer is even more challenging because little is known about the prevalence and nature of adrenal incidentalomas in patients with cancer. The prevailing wisdom is that adrenal tumors found as part of the workup or follow-up of cancer are highly likely to be adrenal metastases and should not count as adrenal incidentalomas (9), yet there is no evidence to support or contradict this concept.

The aim of our study was to determine the prevalence and nature of adrenal abnormalities in radiology reports of cancer patients evaluated for reasons other than suspected adrenal pathology. Understanding the scope of adrenal incidentalomas in patients with cancer will help shape guidelines to manage adrenal masses in this population.

## Materials and methods

### Study participants and eligibility

**Figure 1:**
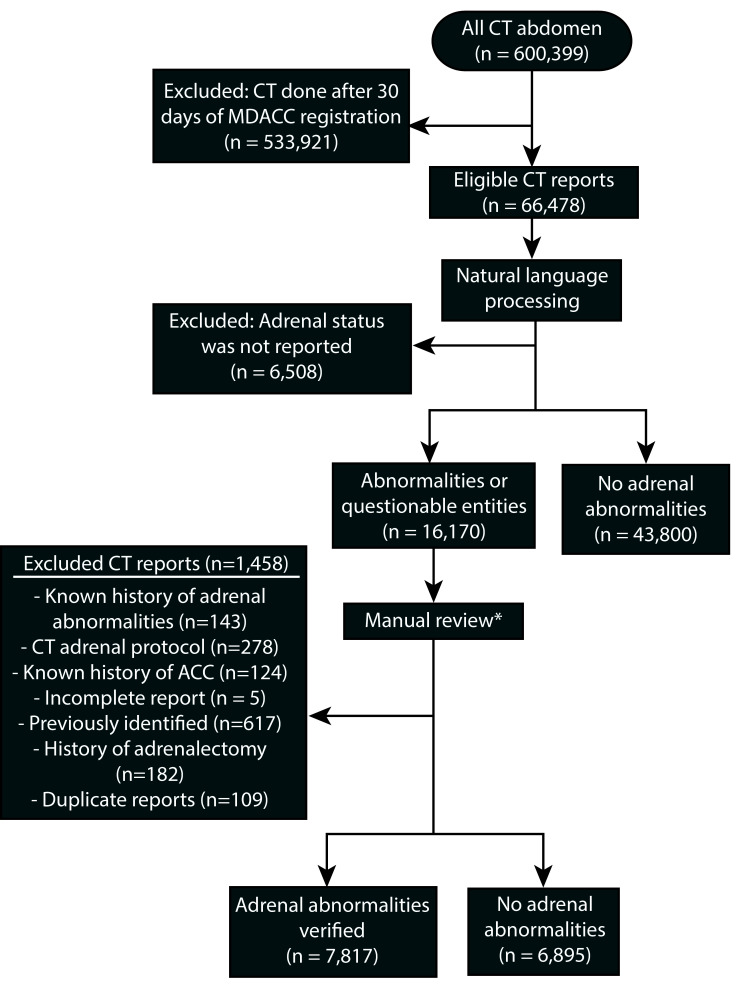
Flow diagram for the steps in identifying the study participants and determining eligibility. * Manual chart review done by three independent abstractors. To assess the inter-rate agreement, 300 charts were reviewed by all the abstractors, yielding a Fleiss kappa of 0.893, which suggests almost perfect agreement between the abstractors.

To identify adrenal abnormalities in CT radiology reports of patients with cancer who were evaluated for reasons other than suspected adrenal pathology at the time of their cancer diagnosis, we queried billing and imaging databases to identify all consecutive patients who underwent abdominal CT imaging at The University of Texas MD Anderson Cancer Center from June 1, 2006, to October 1, 2017. First, the billing database was queried to identify all patients who had current procedural terminology (CPT) codes for abdominal CT, which include abdominal CT with or without pelvis and with or without contrast. We used tumor registry data to cross-reference the selected patients and removed all CT scans completed 30 days or longer after the patient’s registration at MD Anderson Cancer Center to identify only the CTs that were performed as part of baseline investigations. Then, we pulled radiology reports for all the eligible abdominal CT scans from the radiology database. Text mining was used to exclude all reports in which the status of the adrenal glands was not mentioned, and then natural language processing was used to extract adrenal status (**Figure 1**). Specifically, preprocessing and stemming of the complete report’s text, followed by text parsing, was done. Next, two medical doctors weighted identified adrenal entities. This was used to build a multi-class text classifier to classify each report as normal, questionable, or abnormal. The following exclusion criteria were applied for manual review of the radiology reports: 1) history of adrenal abnormalities or adrenocortical carcinoma, 2) history of adrenalectomy, 3) abdominal CT performed as part of an adrenal protocol, 4) incomplete imaging report, 5) abnormality was previously identified on an imaging study, and 6) duplicate imaging report. To assess the accuracy of the natural language processing in successfully classifying a report as normal, we manually sampled about a tenth of the normal reports, and the accuracy of the language processing was reported.

### Report review, data collection and analysis

Patient demographic and cancer information were collected from the databases. Three research team members independently reviewed the reports with questionable or abnormal adrenal findings, as classified by the natural language processing, reclassifying them as either normal or abnormal. The team members also collected the type and laterality of the abnormality and number of lesions. The most prominent abnormality was evaluated if a report contained more than one abnormality. The Fleiss kappa was used to measure the interobserver agreement between the three abstractors (10). The *k* values were interpreted, based on Landis and Koch grouping (11), with a *k* value between 0.81 and 1.00 representing almost perfect agreement, a *k* value between 0.6 and 0.8 representing substantial agreement, a *k* value between 0.41 and 0.6 representing moderate agreement, and a *k* value between 0.21 and 0.4 representing fair agreement. We estimated that the minimum sample size was 281 reports for interobserver agreement analysis, based on 1) the assumption that 281 reports are needed for three raters to achieve the desired lower bound for a two-sided confidence interval for *k* of 0.75 and 2) the anticipated prevalence of the five most frequent abnormalities at a 2-sided 0.05 significance level (12). Therefore, we selected a sample size of 300 reports. Descriptive statistics were used to compare the general characteristics of the cohort. Significance was appraised using chi-square tests for categorical variables and Wilcoxon-Mann-Whitney tests for age, as the age data did not meet the normality assumption. We reported each abnormality’s characteristics.

All statistical analyses were performed using R software (version 3.6.2, The R Foundation, http://www.r-project.org). Main packages used were tm, quanteda, Text2vec, and Stringr. The study was approved by the institutional review board of MD Anderson, which granted waivers of informed consent.

## Results

### Patients’ characteristics

During the study period, 600,399 abdominal CT studies were performed at MD Anderson, and of these, 66,478 were performed within 30 days of the patient’s first presentation to MD Anderson. After applying the exclusion criteria and manually reviewing the questionable and abnormal reports (**Figure 1**), 7,817 (13.4%) CT reports with adrenal abnormalities were identified.

The Fleiss kappa was 0.893, suggesting almost perfect agreement between the abstractors during manual review of the questionable and the abnormal reports. The accuracy of the natural language processing pipeline in identifying normal reports was 97.4%. **Table 1** summarizes our cohort’s general and cancer-related characteristics. The median age for the patients with abnormal reports was 64 years (range, 56-71), which was significantly higher (P<0.001) than the median age for the patients with normal reports (59 years; range, 49-68). Forty-five percent of reported abnormalities were in female patients, and 70.6% of adrenal abnormalities were identified in white patients. Around 22% of abnormalities were identified in patients with primary gastrointestinal tumors, 12.6% in patients with urinary tumors, 8.0% in patients with breast cancer, 7.2% in lung cancer patients, 6.0% in patients with lymphoma, and 6.0% in patients with male genital tract tumors.

**Table 1:** Demographics and clinical characteristics of the patient cohort.

Characteristics	CT finding, n (%)		*P*	Missing (%)
	Adrenal abnormalities	No adrenal abnormalities		
**Total**	7,817 (13.4)	50,695		
**Age, median (IQR), years**	64 (56, 71)	59 (49, 68)	<0.001	0.0
**Sex**			<0.001	0.0
Female	3,498 (44.7)	25,663 (50.6)		
Male	4,319 (55.3)	25,025 (49.4)		
**Race**			0.119	0.1
White/Caucasian	5,516 (70.6)	35,318 (69.7)		
Other	2,299 (29.4)	15,349 (30.3)		
**Cancer type**			<0.001	1.3
Gastrointestinal	1,720 (22.2)	11,365 (22.7)		
Urinary	971 (12.6)	3,701 (7.4)		
Breast	621 (8.0)	6,412 (12.8)		
Lung	558 (7.2)	1,192 (2.4)		
Lymphoma	463 (6.0)	4,420 (8.8)		
Male genital	468 (6.0)	3,608 (7.2)		
Sarcoma	335 (4.3)	2,469 (4.9)		
Gynecological	169 (2.2)	1,754 (3.5)		
Leukemia	98 (1.3)	1,087 (2.2)		
Other	2,334 (29.9)	13,980 (27.6)		

CT, computed tomography; IQR, interquartile range.

### Characteristics of the abnormal adrenal findings

Most of the reports (91.9%) mentioned one type of adrenal abnormality, and 635 (8.1%) reports had more than one type of abnormality. Unilateral abnormalities constituted 5,427 (69.4%) of the cases, of which, 3,810 (70.2%) were identified in the left adrenal gland.

Of the 7,817 (13.4%) reported adrenal abnormalities, adrenal nodularity (3,401 [44%]), adenomas (1,733 [22%]), and metastases (1,337 [17%]) were reported most often. Adrenocortical carcinoma was indicated in only 10 (0.1%) of the abnormal reports, and two of these reports suggested pheochromocytoma as the abnormal finding. **Table 2** shows detailed characteristics of the abnormal adrenal findings.

**Table 2:** Description of adrenal abnormalities in abdominal computed tomography imaging reports obtained within 30 days of registration.

Characteristics	N (%)
**Abnormality**	
Nodularity/Hyperplasia	3,401 (43.5)
Adenoma	1,733 (22.2)
Metastasis	1,337 (17.1)
Mass	662 (8.5)
Invasion by extra-adrenal tumor	283 (3.6)
Myelolipoma	190 (2.4)
Calcification	61 (0.8)
Lymphoma	27 (0.3)
Cyst or abscess	23 (0.3)
Granulomatous disease	21 (0.3)
Hemorrhage	16 (0.2)
Adrenocortical carcinoma	10 (0.1)
Lipoma	9 (0.1)
Angiomyolipoma	8 (0.1)
Pheochromocytoma	2 (0.0)
Hemangioma	1 (0.0)
Other	33 (0.4)
**Laterality**	
Left	3,810 (48.7)
Right	1,617 (20.7)
Bilateral	2,390 (30.6)
**More than one reported abnormality**	
No	7,182 (91.9)
Yes	635 (8.1)

## Discussion

In the past two decades, the advances in medical technology have revolutionized health care. In particular, the wide use of imaging modalities and data sharing have helped many patients but at the same time may have led to unintended consequences in part because of the lack of standardized language to communicate imaging findings. The radiology report remains as the most trusted method to communicate imaging findings between the radiologist and the patient’s care team. Unfortunately, clinical practitioners rarely review the actual images and they often base clinical decisions on reported conclusions.

Our study focuses on the reported incidental adrenal abnormalities in a unique, and very large cohort of cancer patients, based on the radiology reports that were formulated by radiology experts specialized in abdominal imaging.

The results of our study challenges the widespread assumption that majority adrenal masses in cancer patients are metastases from extra-adrenal malignancies (9, 13). In fact, our study results show a higher prevalence of adrenal incidentaloma in CT radiology reports of cancer patients (13.5%) than that reported in the general population (about 4-7%) (3–6). Moreover, our study also showed that most of these reported lesions (83%) were not reported as metastases. The majority of the lesions were reported as nodules (44%), adenomas (22%), and multiple other less prevalent diagnoses (**Table 2**).

These results support the previously reported prevalence of adrenal incidentalomas discovered during follow-up of patients with colorectal carcinoma (6.8%-10.5%) (14, 15). Similarly, most of those reported lesions were non-metastatic. The metastasis rate of 17% in our study falls within the previously reported range of 4% and 26% (14, 15).

Our findings are in line with other retrospective reports that found a plethora of adrenal masses in the general population, including a report that found that 67.9% non-functioning adrenocortical adenomas, 9.4% had adrenocortical carcinoma, 9.4% had ganglioneuromas, 5.7% had pheochromocytomas, 5.7% had adrenal cysts, and 1.9% had myelolipomas (16). One retrospective study compared the short-term clinical and biochemical behaviors of adrenal incidentalomas in patients with and without extra-adrenal malignancy, revealing similar clinical behaviors of adrenal incidentalomas in both types of patients (17).

To our knowledge, there are no large prospective studies on neither the outcomes of cancer patients with adrenal incidentalomas nor the management of adrenal incidentalomas

While there is a plethora of incidental findings (18), little is known about how much these incidental radiological findings should affect the management of adrenal incidentalomas (19). Currently, recommendations by the reporting radiologist appear to influence whether a patient is referred for further investigation (8), and using specific terminology in these reports and utilizing standardized macros that make specific recommendations for hormonal evaluation in patients with adrenal incidentalomas can lead to improved adherence to clinical guidelines (20, 21).

Our study highlights the importance of follow-up for some cancer patients with incidental findings on radiology reports, as follow-up assessment can lead to the diagnosis of other malignancies or diseases that can affect the overall management of these patients.

Thus, our study re-enforces the fact that adrenal tumors found during staging and surveillance of cancer patients should be evaluated with appropriate imaging and biochemical analyses to evaluate the malignant potential and functional status of the adrenal tumors. Cushing syndrome, pheochromocytoma, or primary hyperaldosteronism (if criteria for that is met) should be excluded. The Endocrine Society/American Association of Endocrine Surgery and the European Society of Endocrinology guidelines for the management of incidentally discovered adrenal tumors suggest that image-guided biopsy should be performed for patients with adrenal tumors, and a history of extra-adrenal malignancy should be obtained if the findings would change management decisions and if a differentiation between metastasis and primary cancer cannot be made without a biopsy (22, 23).

The strengths of our study stem from the large number of patients and the unique cancer population. Nevertheless, our study design did not include pathological-radiological correlation. The reported abnormalities reported in this study are not confirmed diagnoses based on the hormonal, histological and follow-up data but rather solely based on the terminology used in the radiology report. This allowed us to highlight the prevalence and spectrum of these incidental adrenal abnormalities as viewed by the oncologists and endocrinologists when reading the radiology report, however, it limited identifying a homogenous specific criteria used by the radiologist to report these abnormalities, considering the retrospective nature of this study. Another limitation is that some of the patients had CT scans performed upon referral rather than at the time of initial diagnosis. Additionally, physicians are more likely to order abdominal CT scans for patients with certain types of malignancies, such as gastrointestinal and genitourinary tumors, than for patients with non-abdominal tumors. This difference might explain the fact that at least 20% of the adrenal abnormalities in our study were identified in patients with primary gastrointestinal tumors, while the actual prevalence of adrenal incidentalomas might be higher in patients with other types of cancer who remain undiagnosed because they are unlikely to get abdominal CT scans. In our study, the high prevalence of adrenal incidentalomas in White patients may have been affected by the demographics of patients referred to our tertiary care cancer center.

Finally, a question could be raised about the cost-effectiveness of detailed evaluation of indeterminate adrenal lesions in cancer patients vs. assuming that these lesions are likely adrenal metastases (24). To answer this question, we suggest an individualized approach to best help every patient and avoid wasting resources. For a patient with a long-expected lifespan and good functional status, an early diagnosis of adrenocortical carcinoma could allow for a complete surgical resection, which is the most important prognostic factor for these tumors. Thus, further follow-up and confirmation of the nature of any incidentally reported adrenal lesion would be warranted, whereas in a patient with an advanced primary malignancy and widespread metastases, the decision to further investigate and likely rule out another primary and likely even more aggressive malignancy should be discussed with the patients and their families. Further prospective studies are needed to validate these results and to determine clinical and radiographic criteria to assist clinicians in determining which patients may benefit from more detailed evaluation based on the radiology reports and the type of workup needed, including further imaging, biochemical evaluation, or biopsy, if indicated.

## Data availability statement

The datasets presented in this article are not readily available because the data are not publicly available and anonymized patient-level data that are compliant with Health Insurance Portability and Accountability Act regulations will be shared upon acquiring MD Anderson Institutional Review Board approval. Requests to access the datasets should be directed to Mouhammed Amir Habra, MD; E-mail: mahabra@mdanderson.org.

## Ethics statement

The studies involving human participants were reviewed and approved by the institutional review board of MD Anderson Cancer Center. The ethics committee waived the requirement of written informed consent for participation.

## Author contributions

MH, KE conceived and designed the study and developed the methods. AQ, SB, OE, AM acquired the data. S-CY supervised statistical analysis. AQ, SB, OE analyzed and interpreted the data. AQ, SB created the figures and tables. MH, SB, AQ, S-CY, KE drafted the manuscript. All authors contributed to the article and approved the submitted version.

## Acknowledgments

We thank Ashli Nguyen-Villarreal, Associate Scientific Editor, and Dawn Chalaire, Associate Director, in the Research Medical Library at The University of Texas MD Anderson Cancer Center, for editing this article.

## Conflict of interest

S-CY received funding from Bristol-Myer Squibb and DepMed, Inc. (now known as Assertio Therapeutics, Inc.) for investigator-initiated studies and was a member of an expert panel for Celgene Corp. MH received research funding from Exelixis Inc and Corcept Therapeutics and was a member of advisory board for HRA pharma and consultant to Orphagen.

The remaining authors declare that the research was conducted in the absence of any commercial or financial relationships that could be construed as a potential conflict of interest.

## Publisher’s note

All claims expressed in this article are solely those of the authors and do not necessarily represent those of their affiliated organizations, or those of the publisher, the editors and the reviewers. Any product that may be evaluated in this article, or claim that may be made by its manufacturer, is not guaranteed or endorsed by the publisher.
